# Quinine and Malaria

**Published:** 1919-01

**Authors:** 


***Quinine and Malaria.*** By G. Pepin. *La Presse Médicale*, September 23, 1918.

The author has devised a method of measuring exactly the quantities of quinine eliminated with the urine. The subjects on whom the experiments were carried out received an intramuscular injection of a solution of 0.800 gr. of quinine hydrochlorid.

13 minutes after the injection 7/20 milligr. of quinine hydrochlorid were detected in 107 cc. of urine.

216.5 milligr. were eliminated on the whole during 48 hours. All tests undertaken to measure the quinine eliminated in other combinations than quinine hydrochlorid always proved unsuccessful.

Furthermore, the author measured the quantities of quinine HCl existing in the urine after absorption of that substance through the stomach, or in the fluid obtained after puncturing cavities existing in the buttocks of patients treated for malaria, or in the feces of a patient to whom intramuscular injections of quinine had been given.

Experiments of that kind might lead to discovery as to whetner or not there is any relation between the chemical and therapeutical activity of quinine, and to determination of the most efficient conditions under which this subtance can be used against malaria.

